# Japan Geriatrics Society “Statement for the use of telemedicine in geriatric care: Telemedicine as a complement to in‐person medical practice”: Geriatric Medical Practice Committee consensus statement

**DOI:** 10.1111/ggi.14490

**Published:** 2022-10-05

**Authors:** The Japan Geriatrics Society Geriatric Medical Practice Committee, Kazushi Nomura, Satoru Ebihara, Yukihiko Ikebata, Hiroyuki Umegaki, Kazuya Ooi, Sumito Ogawa, Tomohiro Katsuya, Yoshio Kobayashi, Takashi Sakurai, Mariko Miyao, Kiyoshi Yamaguchi, Masahiro Akishita

**Affiliations:** ^1^ Nomura Clinic Tokyo Japan; ^2^ Department of Internal Medicine and Rehabilitation Science Tohoku University Graduate School of Medicine Sendai Japan; ^3^ Ikebata Hospital Echizen Japan; ^4^ Department of Community Healthcare and Geriatrics Nagoya University Graduate School of Medicine Nagoya Japan; ^5^ Department of Clinical Pharmacology Suzuka University of Medical Science Suzuka Japan; ^6^ Department of Geriatric Medicine, Graduate School of Medicine University of Tokyo Tokyo Japan; ^7^ Katsuya Clinic Amagasaki Japan; ^8^ Toyano Central Hospital Niigata Japan; ^9^ Department of Prevention and Care Science, Research Institute National Center for Geriatrics and Gerontology Obu Japan; ^10^ At Home Omotesando Clinic Tokyo Japan; ^11^ Fukuro Clinic Todoroki Tokyo Japan

**Keywords:** clinical medicine, community and family medicine, geriatric medicine, others for sociomedical science, sociomedical science

## Abstract

Telemedicine has changed from a way to treat patients with limited access to hospitals to a necessary method of treatment for non‐urgent conditions during the coronavirus disease 2019 pandemic. There are two styles of telemedicine, namely “hybrid medical care” and “gateway medical care,” which take advantage of the characteristics of online medical care and might become important in the near future. During hybrid medical care, a patient and their primary care physician have face‐to‐face medical care while simultaneously being examined by a specialist physician through telemedicine, leading to an overall improvement in the level of local medical care and expansion in the number of treatable diseases. Gateway medical practice is a form of telemedicine used for patients who would otherwise refuse or not receive in‐person medical care to engage in consultation with a physician. Telemedicine allows physicians to determine disease severity and triage patients, while reducing unnecessary home visits, emergency hospitalizations and the spread of infection. Telemedicine is less intense than in‐person medical care, and allows for easier collaboration with other healthcare providers. However, telemedicine is not optimal for conditions requiring a definitive diagnosis and a comprehensive understanding of the patient's medical history. It is limited by the patient's ability to use telemedicine devices, and the risk of accidental treatments and fraud. The use of telemedicine might result in the development of new, online comprehensive geriatric assessment tools and technologies. **Geriatr Gerontol Int 2022; 22: 913–916**.

## Introduction

Telemedicine was developed as a more convenient way to treat patients with limited access to a hospital, such as those located in remote areas or on islands; however, its use increased during the coronavirus disease 2019 (COVID‐19) outbreak.^1^ Telemedicine is limited by the available examination techniques, especially during a patient's first visit, when their medical history is unavailable, or when a definitive diagnosis is required. However, telemedicine allows physicians to determine the severity of a patient's condition, and to triage patients based on symptoms and findings. Furthermore, telemedicine allows for hourly examinations and progress observation that are otherwise challenging through regular medical practice.^2^, ^3^ However, telemedicine is currently only used as a supplement to in‐person medical practices.

Here, we describe two styles of telemedicine practice that might become important in the near future, based on the characteristics of telemedicine, namely, “hybrid medical practice” and “gateway medical practice.” These medical practices will be of importance to the geriatric setting.

Hybrid medical practices include the combination of in‐person medical practice and telemedicine. The use of hybrid medical practices is expected to increase in the future. In hybrid medical practices, a patient and an attending physician are present in the consultation, and telemedicine is used to further consult with a specialist for medical intervention (Doctor to Patient with Doctor). The hybrid medical practice will make specialized medical care, which was previously difficult for family physicians to incorporate, more accessible, increasing the overall level of community medical care, and expanding the number of diseases that can be treated.^4^

Gateway medical practice bridges telemedicine and in‐person medical practice, as it aims to remove healthcare challenges, and create opportunities for casual consultation for patients who do not have family physicians, are unable to obtain medical care or are resistant to receiving medical care. Gateway medical practice is expected to initiate medical care for patients who remain untreated, despite the identification of abnormalities in health checkups or those suspected of having dementia, but cannot undergo examinations.

Several challenges are specific to providing older adults with telemedicine, including the challenge of operating electronic devices and hearing difficulties. Therefore, medical staff are required for support in such cases. Medical practice supporters can visit the patient daily to assess their condition and medication compliance, and assist with medical examinations. These tasks can be easily carried out using the medical practice support staff's electronic devices.^5^, ^6^, ^7^

Telemedicine comprehensive geriatric assessments (CGAs) that include frailty prevention can be used in the future to check the patient's daily living conditions and collect new information regarding the risks of the living environment. Evaluations and preventative measures can be undertaken for previously unknown risk factors. The use of telemedicine might also improve multidisciplinary collaboration efforts.

This statement focused on telemedicine, in which physicians remotely carry out medical examinations and procedures for diagnosis, treatment and management of older patients, and online medical care that includes activities related to nursing care and public health, such as infection control.

## Basic concepts of telemedicine for older adults

1. Telemedicine refers to performing a medical examination, diagnosing patients, prescribing medication and giving patient results in real‐time through Information and Communication Technology between doctors and patients.
  - E‐mail, social media and telephonic medical practices are not included in the category of telemedicine.
2. The physician is primarily responsible for medical practice.
  - The physician carefully judges whether sufficient information can be obtained through telemedicine methods, and whether the information can be used to make an accurate diagnosis. If telemedicine is inappropriate, the online medical practice should be suspended immediately, and in‐person medical practice should be carried out.
3. The first visit is typically carried out by the family physician.
  - Based on a notification issued on 10 April 2020^8^ that encourages “timely and special handling of medical practice using telephones and information and communication equipment amidst the spread of COVID‐19,” it is possible to prescribe medication during the patient's first visit (although narcotics/psychotropic drugs, antineoplastic agents, immunosuppressive agents and other drugs cannot be prescribed, and the maximum number of prescription days is 7).
  - It is important for physicians to stay up‐to‐date with the latest information and notifications, as it is expected that the rules for the first visit of telemedicine will change significantly in the future.
4. The disadvantages and advantages of telemedicine should be well‐understood, as medical practice for older adults is generally carried out during in‐person interactions, and online medical practice complements in‐person treatment.
5. Physicians must comply with the “Guidelines for Proper Implementation of Telemedicine” issued by the Ministry of Health, Labor and Welfare, receive designated training, and carry out telemedicine while showing sufficient understanding.
  - The aforementioned guideline was promulgated in March 2018 (partially revised in January 2022),^9^ and the training application can be found at: https://telemed-training.jp/entry.
  - Telemedicine should comply with the law and the guidelines about the proper handling of personal information in medical and nursing care‐related businesses.

## Disadvantages of telemedicine

1. Telemedicine is unsuitable for diseases requiring a definitive treatment diagnosis.
  - Telemedicine does not allow for several examination methods, including auscultation, palpation and various tests, limiting the amount of information available for and impeding definitive diagnoses.
2. Telemedicine should be used for patients with confirmed medical history.
  - As older adults are at high risk of developing atypical symptoms and multiple diseases, first visits for patients with limited medical history information should only be carried out through online medical practice if the benefits outweigh the risks. In‐person medical practice should be provided as soon as possible.
  - If a serious illness is included in the differential diagnosis, it is necessary to shorten the period until the next medical examination or carry out an in‐person medical practice to track the patient's progress more accurately.
3. Older adults often have difficulty using Information and Communication Technology devices.^5^, ^6^
  - Patients who can operate the devices might be unable to fully utilize them due to impaired hearing.
  - To provide telemedicine for older adults, the medical and nursing care staff or family members should prepare the device settings in advance. A medical support person, such as a family member, a care manager or a visiting nurse, should be present during the telemedicine session.^10^ Telemedicine is preferred over telephone medical practice for patients with impaired hearing.^11^
  - Telemedicine might lead to an increase in consultations for older people with minor and/or mental illnesses because of its easily accessible nature.

## Advantages of telemedicine

1. Telemedicine allows physicians to determine the disease severity and triage patients.
  - If the disease history and patient health status are known, it is possible to use telemedicine to determine the disease severity and to triage the patient using means, such as interviews, pictures and videos of the affected site, and examinations carried out through telemedicine.
  - By examining the patient at home directly through the screen, changes in vitals after exercise and the more natural state of paralysis and dysarthria can be observed.
  - When observing the skin and pharynx for symptoms, such as edema and rash, a sufficient light source and multiple observation angles (including videos) should be used.
2. Telemedicine allows for a reduction in unnecessary home visits.^10^
  - When requesting medical practice due to overnight changes in physical condition in patients in home‐based medical practice, more information is available to determine the required urgency than would be acquired through telephonic re‐examinations.
  - Online interviews effectively prevent omissions in patient information, and allow for improved communication with patients with communication problems or impaired hearing.
3. Telemedicine reduces emergency hospitalizations
  - Sudden illnesses can be managed early without hospital visits, reducing emergency hospitalizations due to sudden changes in the patient's condition.
  - Treatment interruptions are reduced, and sudden changes are prevented.
  - The patient is less hesitant to seek care from a physician.
4. Telemedicine reduces the risk of infection.
  - Contact opportunities are reduced, lowering the risk of the spread of infection.
  - The consultation time and time spent in medical institutions are shortened by online interviews with all patients, including those with suspected infectious diseases.
  - As online medical examinations allow patients to stay at home, examinations of in‐person patients can be prioritized, enabling medical staff to respond according to the number of patients in the hospital (especially for low urgency cases).
5. Telemedicine is less tense than in‐person medical practice, resulting in a sense of security.
  - Telemedicine is highly effective for patients with anxiety and anonymous complaints.^12^
  - Telemedicine is psychologically beneficial for addressing anxiety and pain in terminally ill patients.
  - Telemedicine plays a role as a gateway medical practice for patients who have no history of hospitalizations and are hesitant to go to the hospital, thereby helping patients trust their physicians.
  - Telemedicine reduces the burden associated with going to the hospital (including the caregivers' burden, especially those of patients with dementia).
6. Telemedicine allows for hybrid medical practice.
  - During hybrid medical practice, a patient and an attending physician are present, and telemedicine is carried out by consulting a specialist for medical intervention (Doctor to Patient with Doctor).
  - Hybrid medical practice might improve medical practice in specialized areas with poor access to hospitals and the absence of nearby specialists.
  - In specialized medical practice related to intractable diseases or epilepsy, the remote cooperation medical practice fee can be determined.^13^
7. Telemedicine provides advantages in multidisciplinary collaborations.
  - Sharing telemedicine information with healthcare workers of different disciplines is possible through medical network media with a secure security level.
  - **Nurses:** The act of providing online medical care with the attending physician in the presence of a patient and a visiting nurse (Doctor to Patient with Nurse) allows for changes in oral medications and lifestyle.
  - **Nutritionists:** Online nutritional guidance can be provided, beginning during the first telemedicine visit. Nutrition therapy can be implemented easily for home care recipients with frailty and sarcopenia. Detailed nutritional guidance might be possible through pictures of meals. Furthermore, exercise interventions might be an effective non‐drug therapy.
  - **Pharmacists:** The patient's use of specific medications (such as bisphosphonates), the preparation and storage of injections at home, and how medications are used can be determined during telemedicine. Carrying out regular observations after the start of a new drug with several adverse events might lead to higher effectiveness and safety.
  - **Rehabilitation therapists:** Daily rehabilitation progress can be monitored, improving patient compliance.
8. Telemedicine allows for online CGA, a new stage of CGA.
  - CGAs to evaluate the physical function, mental/psychological state, nutrition, medication and social conditions can be carried out by observing the patient's daily life instead of using a conventional questionnaire.
  - **Physical function:**

- Evaluation of activities of daily living: Observe the patient's movement to the toilet, stair ambulation, and ability to dress oneself and explain the assistance methods required to medical support staff.
- Fall risk assessment (from observations of the patient's movement to the toilet and stairs): Observe the walking conditions and environment where falls are likely to occur (obstacles, steps, handrails and lighting), and explain how to overcome these challenges.
- Carry out a chair standing test and a one‐leg standing test with the support of a caregiver.
  - **Mental/psychological state:** Patients should have a sense of security at home, allowing for more accurate judgments of cognitive function, depression and motivation.
  - **Nutrition:** Check the patient's dietary intake by assessing their meals.
  - **Medicine:** Confirm the accurate use of medications and injection techniques, identify how the medications are managed, and confirm the correct amount of medication remaining.
  - **Social situation (long‐term care system, economic situation and housing environment):**

a Check the sanitary conditions of the kitchen and washroom.
b Check the living environment, including the position of windows and natural light.

9 Telemedicine is an initiative to introduce new technology in the future.

- Telemedicine might lead to using facial expression analysis software using artificial intelligence.
- The implementation of neuropsychological and physical function tests using tablets and smartphones might occur.

## Future issues and directions

1. Precautions must be taken to avoid medical accidents and crimes, such as fraud (physician impersonators and drug resale), during telemedicine.
2. Novel diagnosis and evaluation methods for new diseases that can be carried out during telemedicine might be developed.
3. Telemedicine will allow for developing a new, online CGA.

## Disclosure statement

The authors declare no conflict of interest.

## Data Availability

Data sharing not applicable ‐ no new data generated
